# Anti-Inflammatory and Immunomodulatory Effects of 2-(3-Acetyl-5-(4-Chlorophenyl)-2-Methyl-1*H*-Pyrrol-1-yl)-3-Phenylpropanoic Acid

**DOI:** 10.3390/biomedicines13082003

**Published:** 2025-08-18

**Authors:** Hristina Zlatanova-Tenisheva, Stanislava Vladimirova

**Affiliations:** 1 Department of Pharmacology and Clinical Pharmacology, Medical University of Plovdiv, 4002 Plovdiv, Bulgaria; 2 Department of Organic Synthesis, University of Chemical Technology and Metallurgy, 1779 Sofia, Bulgaria; vladimirova.s@uctm.edu

**Keywords:** pyrrole derivative, carrageenan, lipopolysaccharide, cytokine

## Abstract

**Background:** The pursuit of novel anti-inflammatory agents with enhanced efficacy and safety is crucial. Pyrrole-containing compounds, integral to many NSAIDs, exhibit promising anti-inflammatory properties. Compound **3f** (2-(3-acetyl-5-(4-chlorophenyl)-2-methyl-1*H*-pyrrol-1-yl)-3-phenylpropanoic acid), a pyrrole derivative structurally inspired by the COX-2 selective inhibitor celecoxib, was evaluated for its anti-inflammatory and immunomodulatory effects. **Methods**: Anti-inflammatory activity was assessed in a carrageenan-induced paw edema model in Wistar rats. Compound **3f** was administered intraperitoneally at 10, 20, and 40 mg/kg, either as a single dose or daily for 14 days. Diclofenac (25 mg/kg) served as the reference. Edema volume was measured by plethysmometry. Systemic inflammation was induced by lipopolysaccharide (LPS), and serum levels of the pro-inflammatory cytokine TNF-α and anti-inflammatory cytokines IL-10 and TGF-β1 were quantified by ELISA following single and repeated administration of compound **3f**. **Results:** Single-dose administration of compound **3f** at 20 mg/kg significantly reduced paw edema at 2 h (*p* = 0.001). After 14 days, all tested doses significantly inhibited paw edema at all time points (*p* < 0.001). In the LPS-induced systemic inflammation model, repeated treatment with 40 mg/kg of compound **3f** significantly decreased serum TNF-α (*p* = 0.032). TGF-β1 levels increased significantly after both single and repeated doses (*p* = 0.002 and *p* = 0.045, respectively), while IL-10 levels remained unaffected. **Conclusions:** Compound **3f** exhibits potent anti-inflammatory activity, particularly after repeated dosing, reflected by reduced local edema and systemic TNF-α suppression. The marked elevation of TGF-β1 indicates a potential immunomodulatory mechanism, selectively modulating cytokine profiles without altering IL-10. These findings support compound **3f** as a promising candidate for targeted anti-inflammatory therapy involving cytokine regulation.

## 1. Introduction

The development of new anti-inflammatory agents with improved safety, tolerability, and efficacy remains a priority in biomedical research [1]. Current pharmacotherapies, including nonsteroidal anti-inflammatory drugs (NSAIDs), are often limited by adverse effects or incomplete resolution of inflammation, particularly in chronic conditions where cytokine dysregulation plays a pivotal role. One promising strategy in drug discovery involves structural modification of known pharmacophores to generate novel compounds with targeted activity and improved pharmacological profiles [1,2].

The pyrrole heterocycle is a privileged scaffold in medicinal chemistry, found in a wide array of pharmacologically active compounds, including antimicrobials, antineoplastics, and anti-inflammatory agents [3,4,5,6]. Classic and modern NSAIDs, such as ketorolac, tolmetin, and zomepirac, incorporate pyrrole-based structures and have demonstrated potent COX-inhibitory and anti-inflammatory properties [7,8,9]. Building upon this foundation, we synthesized a pyrrole-containing compound, 2-(3-acetyl-5-(4-chlorophenyl)-2-methyl-1*H*-pyrrol-1-yl)-3-phenylpropanoic acid (**3f**), using celecoxib—a selective COX-2 inhibitor—as a structural prototype. Compound **3f** is part of a larger series of 19 synthesized derivatives designed to explore structure–activity relationships within this chemical class. The design strategy aimed to retain COX-2 selectivity while potentially introducing novel modulatory effects on inflammation-related pathways, particularly those involving cytokines.

Inflammation is a complex biological response to harmful stimuli, involving a tightly regulated interplay of lipid mediators, reactive oxygen species, and cytokines [10]. Pro-inflammatory cytokines such as tumor necrosis factor-alpha (TNF-α) and interleukin-1 (IL-1) initiate and amplify the inflammatory cascade, inducing cyclooxygenase-2 (COX-2) expression, adhesion molecule synthesis, and acute-phase responses [11,12]. Anti-inflammatory and immunoregulatory cytokines, including interleukin-10 (IL-10) and transforming growth factor-beta 1 (TGF-β1), act to resolve inflammation and restore tissue homeostasis [13,14,15]. The balance between these cytokines is critical to determining the outcome of the inflammatory process, and dysregulation is implicated in a wide range of diseases, including rheumatoid arthritis, atherosclerosis, and inflammatory bowel disease [12,16,17].

In this context, targeting cytokine profiles offers a valuable therapeutic avenue beyond traditional prostaglandin inhibition. Several NSAIDs and COX-2 inhibitors have been shown to modulate cytokine production independently of their cyclooxygenase activity, often via inhibition of transcription factors such as NF-κB [18,19,20]. However, such effects are drug- and model-specific, and the cytokine-modulating potential of newer pyrrole-based derivatives remains largely unexplored.

In the present study, we evaluated the anti-inflammatory activity of pyrrole-based compound **3f** in both acute and subacute inflammation models. We employed the carrageenan-induced paw edema model to assess local anti-inflammatory effects, and the lipopolysaccharide (LPS)-induced systemic inflammation model to investigate changes in key circulating cytokines—namely TNF-α, IL-10, and TGF-β1. These cytokines were selected based on their established roles in orchestrating immune responses and mediating the transition from inflammation to resolution. Our objective was to characterize not only the anti-edematous potential of compound **3f** but also its capacity to modulate systemic cytokine levels after single and repeated administration.

## 2. Materials and Methods

All procedures were approved by the Animal Health and Welfare Directorate of the Bulgarian Food Safety Agency, following a positive opinion from the institutional Ethics Committee (Protocol No. 419/20 December 2024).

### 2.1. Reagents

The following reagents were used: diclofenac sodium (75 mg/3 mL ampoules, Hexal AG, Holzkirchen, Germany), 0.9% sodium chloride (Sopharma AD, Sofia, Bulgaria), lambda-carrageenan (Merck, Darmstadt, Germany), lipopolysaccharide (LPS) from *Escherichia coli* strain O55:B5 (Merck, Darmstadt, Germany), and compound **3f** (2-(3-acetyl-5-(4-chlorophenyl)-2-methyl-1*H*-pyrrol-1-yl)-3-phenylpropanoic acid). All substances were dissolved in physiological saline and administered intraperitoneally, except carrageenan, which was injected subplantarly.

**Figure 1:**
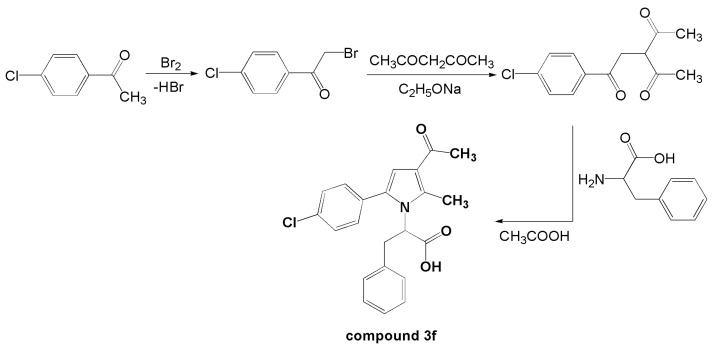
Procedure for synthesis of 2-(3-acetyl-5-(4-chlorophenyl)-2-methyl-1*H*-pyrrol-1-yl)-3-phenylpropanoic acid (**3f**).

Compound **3f** is a pyrrole derivative synthesized via the Paal–Knorr cyclization, with the synthetic route illustrated in Figure 1. Its chemical design, synthesis, and characterization have been described in detail previously by Vladimirova and Bijev [21]. Compound **3f** was purified by recrystallization to a purity greater than 98%, as confirmed by HPLC analysis. Elemental analysis (calculated for C_22_H_20_ClNO_3_: C 69.20%, H 5.28%, N 3.67%; found: C 68.88%, H 5.12%, N 3.34%) and a sharp melting point (163–164 °C) were consistent with the proposed structure. The selection of doses (10, 20, and 40 mg/kg) was based on previous studies involving structurally related pyrrolic compounds. These doses were shown to be pharmacologically relevant and safe in acute oral toxicity tests. Using this established dosing range also facilitates comparison across the broader compound series and supports future quantitative structure–activity relationship (QSAR) analyses by maintaining consistency in pharmacological evaluations.

### 2.2. Experimental Animals

Male Wistar rats (n = 72), 6 weeks old, weighing 150 ± 20 g, were obtained from an accredited breeding facility. Animals were housed under standard laboratory conditions (12/12 h light/dark cycle, controlled temperature, ad libitum access to food and water) and randomly assigned to nine experimental groups (Table 1).

### 2.3. Evaluation of Anti-Inflammatory Activity

To evaluate the anti-inflammatory activity of compound **3f**, paw edema was induced in two experimental settings: following a single dose or after 14 consecutive days of treatment. Baseline paw volume was measured using a plethysmometer (Ugo Basile, Italy), which quantifies paw swelling by detecting changes in volume through fluid displacement. Acute inflammation was induced via subplantar injection of 0.1 mL of 1% carrageenan in saline into the right hind paw. Immediately thereafter, animals received intraperitoneal injections of saline, diclofenac (25 mg/kg), or compound **3f** (10, 20, or 40 mg/kg). Paw volume was recorded at 2, 3, and 4 h post-injection.

Edema was expressed as a percentage increase relative to baseline using the following formula:

Percentage of paw edema = (Vt − Vo)/Vo × 100

Where Vo is baseline paw volume, and Vt is volume at the indicated time point. Reduction in edema versus control was interpreted as an anti-inflammatory effect [22].

### 2.4. Cytokine Profiling in a LPS-Induced Inflammation Model

To evaluate the systemic anti-inflammatory effects of compound **3f**, serum cytokine levels were measured in two experimental settings: following a single dose or after 14 consecutive days of treatment. Only the highest dose (40 mg/kg) was selected for repeated administration to limit animal use and because this dose demonstrated the strongest efficacy in the single-dose setting. Four hours prior to sacrifice, rats in the LPS groups received an intraperitoneal injection of LPS (250 µg/kg). Animals were then euthanized, and blood was collected for serum separation by clotting at 37 °C for 60 min, followed by centrifugation (3000 rpm, 10 min). Serum samples were stored for cytokine analysis [23].

#### ELISA-Based Cytokine Quantification

Serum concentrations of TNF-α (pro-inflammatory), IL-10, and TGF-β1 (anti-inflammatory) were determined using commercial ELISA kits (Platinum ELISA, eBioscience, San Diego, CA, USA), according to the manufacturer’s instructions. Serum dilutions were 1:2 for TNF-α and IL-10, and 1:500 for TGF-β1.

Each cytokine was captured on wells coated with specific monoclonal antibodies, followed by the addition of a peroxidase-conjugated secondary antibody and chromogenic substrate. Optical density was measured at 450/620 nm using a TECAN plate reader. Cytokine concentrations (pg/mL) were interpolated from standard curves.

Assay characteristics:

TGF-β1: Sensitivity: 8 pg/mL; Intra-/inter-assay CV: <3.7%/<8.6%
IL-10: Sensitivity: 1.5 pg/mL; Intra-/inter-assay CV: <5%/<10%
TNF-α: Sensitivity: 11 pg/mL; Intra-/inter-assay CV: <5%/<10%

### 2.5. Statistical Analysis

Data were analyzed using IBM SPSS Statistics v24.0 (IBM, Armonk, NY, USA). Normality was assessed via the Shapiro–Wilk test. For group comparisons, one-way ANOVA was used, followed by Tukey’s post hoc test (homogeneous variances) or Games–Howell test (heterogeneous variances). Results are presented as mean ± SEM. A *p*-value ≤ 0.05 was considered statistically significant.

## 3. Results

### 3.1. Anti-Inflammatory Effect of Compound ***3f***

The anti-inflammatory potential of compound **3f** was evaluated using the carrageenan-induced paw edema model in rats, both after a single administration and following 14 days of repeated dosing. Diclofenac (25mg/kg b.w.) was used as the reference anti-inflammatory agent.

#### 3.1.1. Single Administration

Following a single administration, diclofenac significantly reduced paw edema compared to the saline-treated control group at all three measured time points (2nd, 3rd, and 4th hours). Specifically, paw edema percentages in the diclofenac group were 21.9 ± 2.3%, 14.4 ± 2.9%, and 16.5 ± 3.0%, respectively, with all comparisons showing statistical significance (*p* < 0.05).

**Figure 2:**
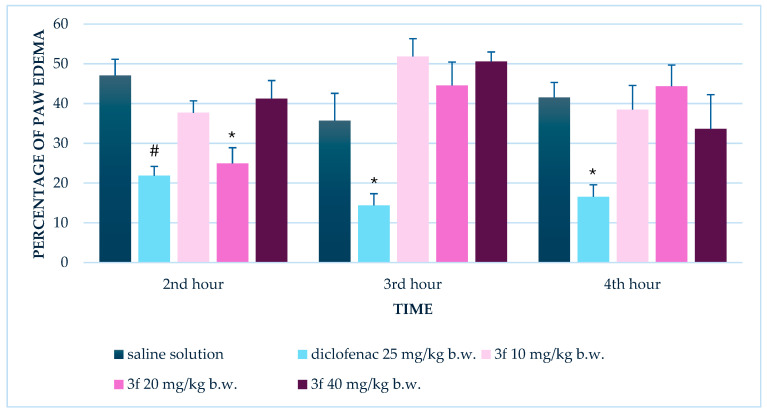
Comparison of edema percentage in the carrageenan-induced paw edema test between control group and groups treated with diclofenac and compound **3f** at doses of 10, 20, and 40 mg/kg b.w. after single administration. * *p* < 0.05 compared to control; # *p* < 0.001 compared to control.

In contrast, compound **3f** at 10 and 40 mg/kg b.w. did not produce significant edema reduction relative to the control at any time point. However, the 20 mg/kg dose of compound **3f** significantly suppressed edema at the 2nd hour (*p* = 0.001) compared to the control; however, this effect was not sustained at the 3rd or 4th hours (Figure 2).

#### 3.1.2. Repeated Administration (14 Days)

Following 14 consecutive days of treatment, diclofenac continued to demonstrate strong anti-inflammatory activity, with paw edema reduced to 14.5 ± 0.9%, 11.2 ± 1.4%, and 7.7 ± 1.3% at the 2nd, 3rd, and 4th hours, respectively (all *p* < 0.001 v.s. control).

**Figure 3:**
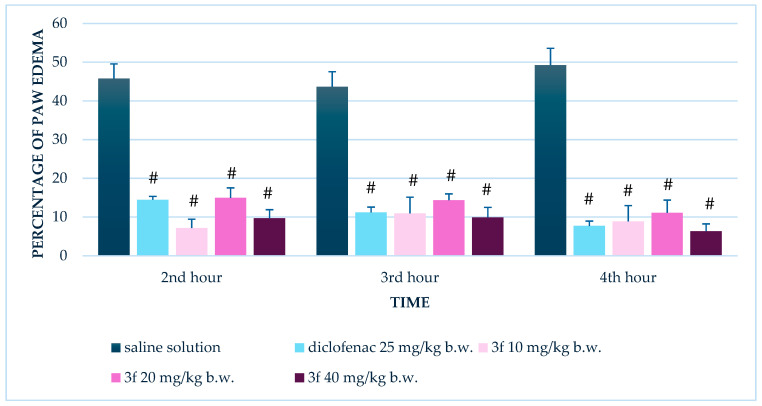
Comparison of edema percentage in the carrageenan-induced paw edema test between control group and groups treated with diclofenac and compound **3f** at doses of 10, 20, and 40 mg/kg b.w. after repeated (14-day) administration. # *p* < 0.001 compared to control.

Notably, compound **3f** exhibited a marked improvement in anti-inflammatory efficacy after repeated dosing. All three tested doses—10, 20, and 40mg/kg b.w.—produced statistically significant reductions in paw edema compared to the control across all time points (*p* < 0.001) (Figure 3).

These findings indicate that compound **3f** displays a time-dependent anti-inflammatory effect, with minimal efficacy after a single administration, but significant activity following repeated dosing. The enhanced response after 14 days suggests cumulative or adaptive mechanisms contributing to its anti-inflammatory action.

### 3.2. Effect of Compound ***3f*** on Serum Levels of TNF-α, IL-10, and TGF-β1

The influence of compound **3f** on systemic cytokine levels was assessed in a LPS-induced inflammation model, both after a single dose and following 14 days of repeated administration at a dose of 40 mg/kg body weight.

#### 3.2.1. Single Administration

After a single administration of compound **3f**, there was a noticeable, though not statistically significant, reduction in serum TNF-α levels (*p* = 0.064). While the decrease suggests a trend toward anti-inflammatory activity, it did not reach the threshold for significance.

In contrast, TGF-β1 levels increased significantly following a single dose of compound **3f** (*p* = 0.002), indicating a potential immunomodulatory or tissue-protective effect.

**Figure 4:**
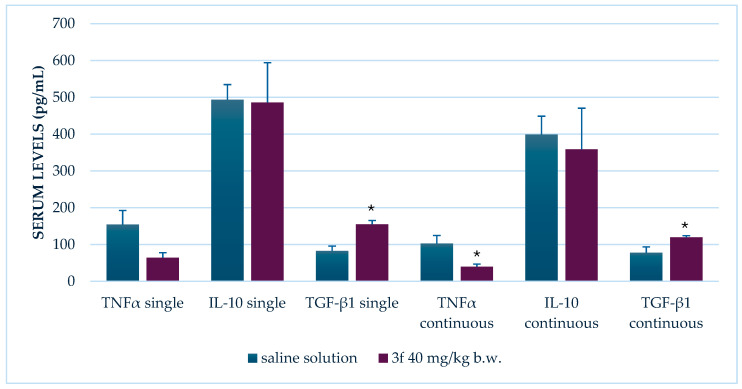
Comparison of serum cytokine levels in pg/mL in LPS-induced systemic inflammation between control group and groups treated with compound **3f** in dose of 40 mg/kg b.w. * *p* < 0.05 compared to control.

Serum levels of IL-10, an anti-inflammatory cytokine, remained largely unchanged after a single administration, with 486 ± 108 pg/mL in the **3f**-treated group compared to 494 ± 41 pg/mL in the control group (*p* = 0.950), suggesting no acute impact of compound **3f** on this cytokine (Figure 4).

#### 3.2.2. Repeated Administration (14 Days)

Prolonged treatment with compound **3f** (40 mg/kg b.w.) over 14 days resulted in a statistically significant decrease in serum TNF-α levels (*p* = 0.032). This sustained suppression confirms the compound’s anti-inflammatory effect upon repeated exposure.

Similarly, TGF-β1 levels were significantly elevated in animals treated with compound **3f** compared to controls (*p* = 0.045). This mirrors the trend observed after a single administration and reinforces the suggestion of a modulatory role for this compound in inflammation resolution or tissue remodeling.

In contrast, IL-10 levels remained unaffected, with mean concentrations of 359 ± 112 pg/mL in the **3f**-treated group versus 399 ± 49 pg/mL in controls (*p* = 0.733), indicating that repeated administration of compound **3f** does not significantly influence this cytokine’s expression (Figure 4).

These findings collectively suggest that compound **3f** exhibits anti-inflammatory effects primarily through TNF-α suppression and TGF-β1 upregulation, particularly evident after prolonged exposure. The lack of effect on IL-10 levels indicates that the compound’s mechanism of action may be independent of this cytokine pathway.

Table 2 presents the precise numerical values for all statistically significant changes observed in paw edema and cytokine levels.

## 4. Discussion

Carrageenan-induced paw edema remains a well-validated experimental model for evaluating the anti-inflammatory potential of pharmacological agents. Initially described by Winter, this acute, non-immune inflammation model is characterized by a biphasic response: an early vascular phase dominated by mediators such as bradykinin, histamine, serotonin, and pro-inflammatory cytokines (e.g., TNF-α) [24], followed by a cellular phase where prostanoids and COX-2-mediated mechanisms predominate [25,26]. Importantly, the suppression of the second phase is considered a hallmark of NSAID activity, especially those with COX-2 inhibitory properties [27]. This model’s translational relevance stems from its ability to predict the clinical efficacy of anti-inflammatory agents, as the doses required to suppress edema in rodents closely approximate those effective in human therapy [28].

Numerous pyrrole-based compounds have previously shown anti-inflammatory activity in this model. Maddila et al. demonstrated that 1,3,4-thiadiazoles bearing a pyrrole nucleus significantly reduced carrageenan-induced inflammation [29]. Mohamed et al. reported pyrrole derivatives with activity comparable to ibuprofen [30], while Di Capua et al. designed fluorinated 1,5-diarylpyrrole ethers with in vivo efficacy [31]. Lavrentaki et al. synthesized eight N-substituted pyrrole-indazole hybrids, with two showing a notable reduction in edema [32]. Similarly, Pasha et al. developed aryl (4-aryl-1*H*-pyrrol-3-yl)(thiophen-2-yl) methanone analogues and identified compound **PY5** as effective in suppressing inflammation in vivo [33]. Wang et al. designed 7*H*-pyrrolo [2,3-d]pyrimidine compounds, with compound **N3b** significantly attenuating both acute and chronic inflammation [34]. Together, these findings support the pyrrole scaffold as a privileged structure for anti-inflammatory drug development.

In our study, we evaluated compound **3f**, a molecule structurally characterized as 2-(3-acetyl-5-(4-chlorophenyl)-2-methyl-1H-pyrrol-1-yl)-3-phenylpropanoic acid. This compound integrates multiple pharmacophores associated with anti-inflammatory activity: a pyrrole core, a 4-chlorophenyl substituent, a ketone (acetyl) group, a methyl group at position 2, and a propionic acid chain terminated with a phenyl ring. The chlorinated aromatic moiety may enhance lipophilicity and membrane permeability [35], while the phenyl ring is known to interact with a variety of biological targets, including enzymes and receptors involved in inflammation [2,36]. The propionic acid moiety mimics structures found in NSAIDs (e.g., ibuprofen), suggesting potential COX inhibition [37]. Moreover, the electron-withdrawing chlorine and carbonyl functionalities could affect binding affinity to enzymatic pockets or influence redox-sensitive pathways involved in cytokine modulation [38].

Compound **3f** was evaluated for anti-inflammatory efficacy in the carrageenan-induced paw edema model, both after a single dose and following repeated administration over 14 days. As expected, the reference drug diclofenac, a relatively selective COX-2 inhibitor, significantly suppressed paw edema at all measured time points following a single dose. However, compound **3f** did not exhibit comparable activity upon a single administration. A transient effect was observed only at the 20 mg/kg dose at the 2 h time point. These findings are consistent with previous literature on selective COX-2 inhibitors, such as celecoxib and rofecoxib, which display minimal acute anti-inflammatory activity but become effective after repeated administration [39]. This delayed efficacy may reflect a time-dependent accumulation or adaptive pharmacodynamic mechanism.

Strikingly, after 14 days of repeated dosing, all three tested doses of compound **3f** (10, 20, and 40 mg/kg) produced significant reductions in carrageenan-induced paw edema, with inhibitory effects comparable to or surpassing those of diclofenac. These findings strongly suggest that compound **3f**’s anti-inflammatory properties are cumulative and may involve stable or irreversible interactions with inflammatory targets, possibly via COX-2 modulation or cytokine pathway regulation. Furthermore, the similar degree of edema suppression across all three doses after prolonged administration suggests saturation of the target effect and potential nonlinear pharmacokinetics.

Given the increasing recognition of cytokines as pivotal mediators in inflammation and resolution, we further assessed the effects of compound **3f** on serum levels of TNF-α, IL-10, and TGF-β1 in a LPS-induced systemic inflammation model. This model simulates systemic immune activation via Toll-like receptor 4 engagement on macrophages and dendritic cells, leading to a cascade of cytokine release (e.g., TNF-α, IL-1β, IL-6), nitric oxide production via iNOS, and COX-2 activation [40,41].

Pyrrole derivatives have previously demonstrated anti-inflammatory efficacy in LPS models. Jung et al. reported that pyrrole-2,5-dione analogues suppressed NO and cytokine release in BV2 microglial cells [42]. Pandey et al. found several penta-substituted pyrrole–hydroxybutenolide hybrids to potently inhibit TNF-α in LPS-stimulated THP-1 monocytes [43]. Similarly, Paprocka et al. showed that pyrrole-2,5-dione compounds suppressed TNF-α and IL-6 in PBMCs [44], while Xu et al. isolated pyrrole alkaloids from *Curvularia lunata* that inhibited NO and IL-6 and promoted IL-10 secretion [45]. These findings highlight the capacity of pyrrole-based compounds to modulate both pro- and anti-inflammatory cytokine pathways.

Following a single dose of compound **3f** (40 mg/kg), TNF-α levels were reduced by more than half compared to the LPS control group; however, this decrease did not reach statistical significance. This result indicates a potential trend toward TNF-α suppression, which became more pronounced upon repeated exposure. After 14 days of treatment, serum TNF-α levels were significantly reduced, confirming that compound **3f** mediates anti-inflammatory effects through inhibition of this key pro-inflammatory cytokine. TNF-α plays a central role in orchestrating the inflammatory cascade by promoting the release of other cytokines such as IL-6 and IL-8, driving leukocyte recruitment, and activating transcription factors like NF-κB [46,47]. Therefore, the sustained suppression of TNF-α by compound **3f** may underlie its therapeutic potential in chronic inflammatory states.

Interestingly, serum IL-10 levels remained unchanged after both single and repeated dosing. IL-10 is a potent anti-inflammatory cytokine that downregulates Th1 responses and inhibits monocyte/macrophage activation [47]. While some NSAIDs modulate IL-10 production [48], our findings suggest that compound **3f** does not rely on this pathway, and its mechanism of action may be IL-10-independent. This further supports a selective modulation of the pro-inflammatory axis rather than a broad-spectrum immunosuppression.

Conversely, TGF-β1 levels were significantly elevated following both single and repeated administration of compound **3f**. TGF-β1 is a pleiotropic cytokine with context-dependent effects, acting as both a suppressor of inflammatory responses and a regulator of tissue remodeling [49,50]. It inhibits the activation and proliferation of T and B lymphocytes and suppresses pro-inflammatory cytokine synthesis [51]. Its consistent upregulation in our study suggests a contributory role in the resolution of inflammation and possible tissue-protective effects associated with compound **3f**. Importantly, TGF-β1 has been implicated in shifting the immune balance toward tolerance and regeneration, making its elevation particularly relevant in chronic or relapsing inflammatory conditions.

Comparable studies have shown that NSAIDs may exert cytokine-modulatory effects independent of prostaglandin inhibition [52]. For example, celecoxib has been reported to reduce levels of IL-1β, IL-6, and TNF-α in osteoarthritic tissues [53], while piroxicam has also been shown to reduce IL-1, IL-6, and TNF-α production in healthy human volunteers [54]. In a spinal cord injury model, meloxicam significantly reduced levels of pro-inflammatory cytokines, whereas methylprednisolone and diclofenac had limited impact on anti-inflammatory cytokines [55]. Similarly, aspirin and celecoxib reversed cytokine dysregulation in myocardial hypertrophy [56], and celecoxib has shown efficacy in TGF-β-driven disease states [57], likely through NF-κB inhibition [58]. However, the immunomodulatory effects of NSAIDs are variable and influenced by compound-specific properties.

Our findings position compound **3f** as a promising anti-inflammatory candidate with a delayed but robust effect profile. Its efficacy appears to be driven by suppression of TNF-α and induction of TGF-β1, without significantly affecting IL-10. The lack of acute activity after single dosing but strong suppression after repeated administration may reflect the compound’s requirement for cumulative exposure to achieve effective cytokine modulation or intracellular signaling interference. Importantly, this study demonstrates that targeted cytokine modulation—specifically TNF-α inhibition combined with TGF-β1 upregulation—can be achieved with a small-molecule pyrrole derivative. Such a mechanism offers the potential for developing safer, more selective anti-inflammatory drugs compared to conventional NSAIDs, minimizing broad immunosuppression while promoting inflammation resolution. The differential cytokine modulation profile distinguishes compound **3f** from traditional NSAIDs and supports further investigation into its potential as a selective immunomodulatory agent.

In conclusion, compound **3f** exerts significant anti-inflammatory effects in both local (carrageenan) and systemic (LPS) models. These effects are associated with TNF-α inhibition and TGF-β1 upregulation after repeated administration, highlighting its potential utility in chronic inflammatory disorders where cytokine dysregulation plays a central pathogenic role. Further mechanistic studies are warranted to elucidate the precise molecular targets of compound **3f** and its long-term safety profile.

## 5. Conclusions

Compound **3f** demonstrated a delayed but significant anti-inflammatory effect, primarily evident after 14 days of administration. This effect was marked by a statistically significant reduction in serum TNF-α levels, a central pro-inflammatory cytokine. Notably, compound **3f** did not alter IL-10 concentrations, indicating that its mechanism of action does not involve broad anti-inflammatory suppression via IL-10 pathways. In contrast, it significantly elevated serum TGF-β1 levels following both single and repeated dosing, suggesting an immunomodulatory role and possible promotion of inflammation resolution or tissue repair processes. These cytokine-specific effects—TNF-α inhibition and TGF-β1 upregulation—underscore the selective immune-modulating potential of compound **3f**. Given the localized nature of the carrageenan-induced inflammation model versus the systemic impact reflected in serum cytokine levels, the need for prolonged administration may reflect the time-dependent establishment of systemic cytokine modulation. Overall, the findings support further investigation into compound **3f** as a cytokine-targeted therapeutic candidate for chronic inflammatory diseases.

## 6. Limitations

This study has several limitations. First, the anti-inflammatory activity of compound **3f** was evaluated exclusively in acute rodent models (carrageenan-induced paw edema and LPS-induced systemic inflammation), which may not fully replicate chronic or complex human inflammatory conditions. Second, only male Wistar rats were used, limiting the generalizability across sexes and species. Third, the mechanisms underlying the observed time-dependent effects remain speculative, as molecular targets and pharmacokinetics of compound **3f** were not directly assessed. Fourth, cytokine profiling was limited to TNF-α, IL-10, and TGF-β1, excluding other relevant mediators that could further elucidate the compound’s immunomodulatory profile. Finally, while doses were selected based on related compounds and acute toxicity data, no detailed pharmacokinetic or dose-response studies were performed to optimize dosing regimens. Future research should address these gaps, including chronic inflammation models, sex-based differences, mechanistic studies, and comprehensive cytokine panels.

## Figures and Tables

**Table 1:** Experimental groups, treatment conditions, and test models.

Group	Treatment	Paw Edema Model	LPS-Induced Inflammation Model	n
1	Saline (carrageenan control)	Yes—single and repeated administration	No	8
2	Saline (LPS single-dose control)	No	Yes—LPS administered on day of experiment	8
3	Saline (LPS repeated-dose control)	No	Yes—LPS administered on day of experiment after 14 days of saline treatment	8
4	Diclofenac 25 mg/kg	Yes—single and repeated administration	No	8
5	Compound **3f** 10 mg/kg	Yes—single and repeated administration	No	8
6	Compound **3f** 20 mg/kg	Yes—single and repeated administration	No	8
7	Compound **3f** 40 mg/kg	Yes—single and repeated administration	No	8
8	Compound **3f** 40 mg/kg (single administration) + LPS	No	Yes—LPS administered on day of experiment	8
9	Compound **3f** 40 mg/kg (repeated administration) + LPS	No	Yes—LPS administered on day of experiment after 14 days of treatment	8

Note: All doses expressed as mg/kg body weight. For LPS groups, LPS (250 µg/kg, i.p.) was administered 4 h prior to sacrifice on the day of cytokine measurement.

**Table 2:** Effects of compound **3f** on paw edema percentages and serum cytokine levels in LPS-induced inflammation after single and repeated administration.

Parameter	Dose (mg/kg)	Time (h)/ Measurement Point	Control (Mean ± SEM)	Treated (Mean ± SEM)	Significance (*p*)	Administration
Paw Edema (%)	20	2	47.1 ± 4.1	25.0 ± 3.9	0.001	Single dose
	10	2	45.8 ± 2.9	7.2 ± 2.3	<0.001	Repeated (14 days)
	10	3	40.5 ± 3.9	11.0 ± 4.2	<0.001	Repeated (14 days)
	10	4	41.0 ± 4.0	8.9 ± 4.1	<0.001	Repeated (14 days)
	20	2	45.8 ± 2.9	14.9 ± 2.6	<0.001	Repeated (14 days)
	20	3	40.5 ± 3.9	14.4 ± 1.6	<0.001	Repeated (14 days)
	20	4	41.0 ± 4.0	11.1 ± 3.3	<0.001	Repeated (14 days)
	40	2	45.8 ± 2.9	9.7 ± 2.2	<0.001	Repeated (14 days)
	40	3	40.5 ± 3.9	9.9 ± 2.6	<0.001	Repeated (14 days)
	40	4	41.0 ± 4.0	6.4 ± 1.9	<0.001	Repeated (14 days)
TNF-α (pg/mL)	40	4 h post-LPS	155 ± 38	64 ± 14	0.064 (trend)	Single dose
	40	4 h post-LPS	103 ± 22	40 ± 7	0.032	Repeated (14 days)
TGF-β1 (pg/mL)	40	4 h post-LPS	83 ± 13	155 ± 10	0.002	Single dose
	40	4 h post-LPS	78 ± 16	119 ± 5	0.045	Repeated (14 days)

Control values for paw edema represent measurements in saline-treated animals.

## Data Availability

All data generated or analyzed during this study are included in this article.

## Abbreviations

The following abbreviations are used in this manuscript:

NSAIDs	Non-steroidal anti-inflammatory drugs
COX	Cyclooxygenase
LPS	Lipopolysaccharide
TNF-α	Tumor necrosis factor alpha
IL-10	Interleukin 10
TGF-β1	Transforming growth factor beta 1
NF-κB	Nuclear factor kappa B
ELISA	Enzyme-linked immunosorbent assay
HPLC	High-performance liquid chromatography
